# Very low neighbourhood income limits participation post stroke: preliminary evidence from a cohort study

**DOI:** 10.1186/s12889-015-1872-5

**Published:** 2015-06-04

**Authors:** Mary Egan, Lucy-Ann Kubina, Claire-Jehanne Dubouloz, Dorothy Kessler, Elizabeth Kristjansson, Michael Sawada

**Affiliations:** School of Rehabilitation Sciences University of Ottawa, 451 Smyth Road, K1H 8M5 Ottawa, ON Canada; School of Psychology, University of Ottawa, 136 Jean-Jacques Lussier, K1N 6N5 Ottawa, ON Canada; Department of Geography, University of Ottawa, 60 Université, K1N 6N5 Ottawa, ON Canada; Bruyere Research Institute, 43 Bruyere St, K1N 5C8 Ottawa, ON Canada

**Keywords:** Human activities, Stroke, Poverty

## Abstract

**Background:**

Neighbourhood income level is associated with the incidence of stroke and stroke-related mortality. It has also been linked to receipt of appropriate services, post discharge motor recovery and functional status following a stroke. We examined the impact of neighbourhood income on participation among community-dwelling stroke survivors during the two years following the stroke.

**Methods:**

Secondary analysis of data from a prospective cohort study. Participants were 67 individuals who were treated in acute care or rehabilitation following a first ever stroke, and were discharged to the community with FIM™ scores of at least 3 for comprehension, memory and problem solving. On this functional independence measure, these scores indicate that assistance is needed with related tasks up to 50 % of the time. Participation at 6, 9, 12, 18 and 24-months post stroke was measured using the Reintegration to Normal Living Index (RNLI). Income was measured by median neighbourhood annual family income according to postal code. The impact of very low neighbourhood income (median family income $20,000 Cdn or less) on participation at each follow-up period was determined controlling for potential confounders.

**Results:**

Six (9.0 %) of the participants lived in very low-income neighbourhoods. These participants had average RNLI scores approximately 25 % lower at each follow-up period. While there was a trend for increasing participation with time among those in higher income neighbourhoods, this was not seen among very low-income neighbourhood participants. Very low me neighbourhood income had an independent effect on participation after controlling for discharge FIM™, 2-min walk test, gender, self-rated health, age, and emotional well-being at all follow-up periods.

**Conclusions:**

Our results indicate that very low neighbourhood income is linked with decreased participation during the first two years following stroke. Our findings indicate the need for further investigation of this relationship, and the importance of close follow-up of stroke survivors living in very low-income contexts.

## Background

Stroke is a major contributor to chronic disability in adult populations [1]. Engagement in personally valued activities is critical for a satisfying life after stroke [2–4]. Such engagement has been labeled “participation in life situations” in the World Health Organization’s model of Functional Health and Disability (ICF) [5].

Chronic deficits in participation are extremely common following stroke. Mayo and colleagues found that approximately half of their population-based sample of 434 individuals who had survived a stroke experienced great difficulty participating in meaningful personal and social activities 6 months following stroke [6]. These findings were corroborated by Harwood et al who found that among 141 individuals one year post-stroke “inability to occupy one’s time in a manner appropriate to one’s age, sex and background was by far the most problematic of all areas assessed” (p. 827), with 76 % of respondents noting extreme, severe or considerable problems [7]. The results of the North East Melbourne Stroke Incidence Study were remarkably similar; here 75 % of 226 individuals one year post-stroke reported having difficulty participating in such activities [8].

Evidence from studies of community-dwelling stroke survivors reveals that problems with participation cannot be completely predicted from disability levels. Notably, even among survivors of mild stroke, the majority reports problems in personally valued activities [9]. Less than half of the variation in participation can be accounted for by differences in endurance [10], age [11, 12], comorbidity [12], impairment [11, 12], affect [12], and perceived control [11, 13].

While earlier models of disability viewed difficulties with participation as arising primarily from injury or illness and resulting impairments, the ICF recognizes the importance of contextual factors as potential facilitators of engagement in complex activities. Such factors play an important role in facilitating or limiting participation in life situations. Previous examination of contextual factors has been limited to perceived obstacles in the physical environment [11] and social support [11, 13, 14]. While neighbourhood income levels are not specifically mentioned as a potential contextual factor in the ICF model, their possible impact on participation can be easily conjectured from Bernard’s conceptualization of the relationship between health inequities and neighbourhood income [15].

In Bernard’s theoretical model, neighbourhoods differ in their access to and quality of resources related to health, as well as residents’ agency to maintain or improve resource access. Access to resources is determined through characteristics of the physical environment (such as green space, air quality, community recreation facilities) and rules of the social environment (related to economic rules, institutional rules and informal reciprocity rules). These contextual issues take on even greater importance in situations where people are less mobile, which is often the case when disability is present. When applied to participation following a stroke, people living in lower income neighbourhoods may be further away from green spaces where they could engage in personally valued activities; routes to community recreation facilities may be less walkable; there may be higher levels of pollution and perceived crime; and community organizations that could mobilize for better access may be less stable, wealthy and politically connected [16].

Neighbourhood income has been linked to stroke incidence, mortality and severity, as well as to receipt of optimal post stroke care. A higher risk of stroke has been observed among people in poorer neighbourhoods compared to those living in the most affluent neighbourhoods of two Canadian cities [17, 18]. Stroke mortality is affected by neighbourhood income as well. Among all patients admitted for stroke in Ontario 1994-1997 each $10 000 (Cdn) increase in median neighborhood income was associated with a 9 % reduction in the hazard of death at 30 days (adjusted hazard ratio 0.91, 95 % CI 0.87 to 0.96) and a 5 % reduction in the hazard of death at 1 year (adjusted hazard ratio 0.95, 95 % CI 0.92 to 0.99) [19]. However, in a follow-up study using data from 2002-2005, increased 1-year and 3-year mortality rates were seen only among people in the two lowest neighbourhood income quintiles [20]. Similarly, in Scotland, Weir noted a threshold, rather than a gradient effect, of neighbourhood income and death and institutionalization due to stroke, with people from the 2 poorest of 7 neighbourhoods more likely to experience death or institutionalization, after controlling for potential confounders including age, gender, diabetes, blood pressure and function on admission [21].

While there appears to be a clear association between death and low neighbourhood income, the link between stroke severity and low neighbourhood income seems to vary. Stroke survivors from lower income neighbourhoods in Glasgow tended to have experienced more severe strokes, as well as more frequent hospital readmission for vascular-related issues during 24 months following stroke [22]. However, in Ontario, Canada, stroke severity did not vary by neighbourhood income quintile [20].

Differences in post stroke care have been observed according to neighbourhood income, even in regions with universal health care. Between 1994 and 1997, Canadian stroke patients in the lowest income quintile neighbourhoods were less likely than those in the highest to receive in-hospital physiotherapy (58 % versus 61 %, *P‹*0.001), occupational therapy (36 % versus 47 %, *P‹*0.001), and speech-language pathology (21 % versus 28 %, *P‹*0.001) [19]. In a follow-up study using data from 2003-2008 differences in proportions of patients receiving therapy had disappeared, but other differences were present. People from the lowest income quintile neighbourhoods were less likely to have received care on a stroke rehabilitation unit, to have been cared for by a neurologist or to have been referred to a secondary stroke prevention clinic [23]. Saposnik, also working with Canadian data, found those in lower income quintiles tended to be treated in hospitals with lower volumes of stroke patients [24], an important consideration as hospitals with higher volumes of stroke patients tend to have lower stroke-related mortality rates [25].

No studies could be found that compared functional recovery by neighbourhood income. However, personal income and functional recovery have been examined. In Dhamoon and collegues’ study of stroke survivors in Manhattan, personal income was estimated using medical insurance status (Medicaid or uninsured versus privately insured) and basic activities of daily living were assessed at 6 months and every year post stroke. Functional status was similar for the two groups for the first 2 years. However, at 3-5 years post stroke, those with lower socioeconomic status demonstrated a declining trajectory, while those with higher socioeconomic status showed increasingly better functional abilities [26].

We found initial evidence of the importance of neighbourhood income to participation in the two years following stroke in a cohort study of the potential reciprocal effects of participation on physical and emotional well-being [27]. Median neighbourhood income was included in the data set when the research assistant (LAK) hypothesized that income status was a potentially important factor that we had not considered. Using hierarchical linear regression, we noted that median neighbourhood income modified the association between participants’ self-rated health status and participation. At higher neighbourhood income levels, better self-rated health was associated with improved participation. However, at lower neighbourhood income levels, this association disappeared. The objective of the current study was to more closely examine the potential effects of low neighbourhood income on post stroke participation.

## Methods

We carried out a secondary analysis of data from a prospective cohort [27]. Participants comprised patients who had been discharged to a non-institutional setting from a hospital or a rehabilitation centre prior to discharge back to a non-institutional setting following treatment for a first stroke. Inclusion criteria included the ability to communicate in English or French; FIM™ [28] scores at rehabilitation discharge of at least 3 for comprehension, memory and problem-solving; residence within the City of Ottawa and; potential to be followed for 24 months (no life-threatening comorbidities or plans to move from the region). FIM™ cut-off on scores on this popular standardized tool correspond with needing help not more than 50 % of the time to carry out activities requiring comprehension, memory and problem-solving. This cut-off score was selected to help ensure participants could respond to assessment tools. Recruitment took place between March 2008 and February 2010 from one acute care stroke unit and two stroke rehabilitation units.

Date of birth, stroke location and type and stroke severity as indicated by the FIM™ at discharge were extracted from the participants’ medical records, and median family income for the neighbourhood (based on postal codes) in which each participant lived was obtained from 2006 census data available from Statistics Canada.

Additional data was collected at 6, 9, 12, 18 and 24 months post stroke for the primary outcome, participation, and several potential confounders. The Reintegration to Normal Living Index (RNLI) was used to measure participation [29]. This is an 11-item scale and includes questions regarding important daily activities such as mobility in the home, mobility in the community, taking trips, self-care, work activities (including volunteering, housework and studying), recreational activities, social activities, and family activities. All questions are worded to take into consideration how satisfactory the present situation was to the individual (for example: “I am able to participate in recreational activities as I want to”), thus reflecting engagement in personally-valued activities. A 10-point Likert scale was used in lieu of the original visual analogue scale, as is more appropriate post stroke.

The following tools were used to measure three potential confounders of the relationship between income and participation: the General Well-Being Schedule for (emotional well-being) [30], the 2-min walk test for cardiovascular status and mobility [31], and the General Self-Rating of Health question for overall health [32].

Data analysis was carried out as follows. First, for each time period, RNLI scores were plotted against neighbourhood income to determine if there was an evident low-income cut-point. Then, the average RNLI score at each time point was calculated, along with the mean RNLI score for participants above and below the cut-point. After this, bivariate correlations between RNLI scores and the potential confounders gender, age, emotional well-being, perceived health, walking ability and impairment as measured by FIM™ at discharge were calculated. Then, four linear regression models were developed to determine the impact of very low neighbourhood income on RNLI score, controlling for the previously mentioned potential confounders which demonstrated a bivariate correlation with RNLI of 0.25 or greater [33].

Finally, in addition to the regressions at each time point, a generalized estimating equation (GEE) with an identity link was estimated on all of the data. A GEE specifies a covariance structure for the within-subject dependencies to deal with the usual error non-independence that occurs with repeated measures data. Several different covariance structures were fit and compared on the basis of information criteria (QIC and QICC, both in smaller-is-better form). The first structure was AR, which specifies that the correlations decay at an exponential rate as time passes. The second was an exchangeable structure (also known as compound symmetry), which assumes equal covariances between each pair of time points. The final pattern was unstructured, which makes no assumptions and separately estimates each element of the covariance matrix. The two QIC favored the exchangeable structure, and these are the results that are presented below.

Ethics review was carried out by the Research Ethics Boards of the Elisabeth Bruyère Hospital, the Ottawa Hospital and the University of Ottawa.

## Results

During the data collection period, 192 individuals met the study eligibility requirements. Of these, four could not be reached after repeated calls, 121 declined, and 67 participated (34.9 % participation rate). One participant died during the follow-up period. One participant was not followed at 12, 18 and 24-months, and five participants were not followed at 24-months due to late enrolment in the cohort.

Characteristics of the participants are provided in Table 1. Only six of the participants lived in very low-income neighbourhoods. A higher proportion of the very low-income neighbourhood participants were male (83.3 %) compared to participants in the other neighbourhood group (55.7 %). However, there were no statistically significant differences between the very low-income neighbourhood and other income neighbourhood participants in terms of gender, or age, living arrangements, or occupational status.

**Table 1:** Participant characteristics

	All (n = 67)	Very low income (n = 6)	Other income (n = 61)	p-value
Number of men (%)	39 (58.2)	5 (83.3)	34 (55.7)	0.19
Mean age (SD), range	64.8 (13.3), 33-88	63.3 (14.6), 44-82	64.5 (13.1), 33-88	0.81
Living situation				0.06
Lives alone (%)	13 (19.4)	2 (33.3)	11 (18.0)	
With spouse (%)	46 (68.7)	2 (33.3)	44 (72.1)	
Other (%)	8 (11.9)	2 (33.3)	6 (9.8)	
Occupational status				0.67
Retired pre-stroke (%)	29 (43.3)	3 (50.0)	26 (42.6)	
Retired post stroke (%)	7 (10.4)	0 (0)	7 (11.5)	
Disability or sick leave (%)	18 (26.9)	2 (33.3)	16 (26.2)	
Working full or part-time (%)	7 (10.4)	0 (0)	6 (9.8)	
Homemaker (%)	3 (4.5)	0 (0)	3 (4.9)	
Unemployed (%)	3 (4.5)	1 (16.7)	2 (3.3)	
Mean discharge FIM™ (SD), range	111.3 (13.6), 59-126	107.3 (11.7)	111.7 (13.8)	0.82

*p*-values of t-tests or chi square test

Plotting of the RNLI data against income demonstrated that a median neighbourhood income of $20,000 seemed to be an evident cut point. No participants under this neighbourhood income level attained RNLI scores higher than approximately 90 out of a possible 110 points, while some participants at all other income levels obtained maximum scores. This cut point made logical sense as well, as it corresponds approximately with an annual income at minimum wage levels in Ontario and levels of disability support payments for persons without dependents, as well as federal government low income cut-off levels for single adults [34].

Changes in RNLI levels from six months to 24 months post stroke on average rose gradually throughout the follow-up period. However, when the cohort was separated according to income, the increase was observed only in the higher income group. Participants in the very low-income group began with lower RNLI scores and these scores did not increase substantially throughout the follow-up period (Table 2).

**Table 2:** RNLI scores by income level

	6 m	n	9 m	n	12 m	n	18 m	n	24 m	n
All participants	83.4	67	85.5	63	86.8	65	87.8	61	89.6	55
(SD)	(18.9)		(18.6)		(17.3)		(18.6)		(18.9)	
Income > $20,000	85.8	61	87.6	57	88.8	60	89.7	56	92.3	50
(SD)	(17.0)		(17.9)		(15.7)		(17.1)		(16.9)	
Income ≤ $20,000	59.8	6	64.7	6	62.2	5	66.0	5	62.4	5
(SD)	(22.7)		(12.5)		(19.6)		(22.9)		(17.4)	

Among bivariate correlations between RNLI levels and potential confounders for the relationship between income and participation, only emotional well-being, meters walked, gender and perceived health status demonstrated correlations of 0.25 or better. These variables, along with income level, were entered into multivariate models of RNLI levels at each of the five follow-up time periods. After controlling for these variables, very low income remained a significant predictor of RNLI level at 6, 9 and 12 months, but not 18 and 24 months post stroke (Tables 3, 4, 5, 6, 7). The models predicted between 0.47 and 0.58 of the variability in RNLI level.

**Table 3:** Multivariate model of RNLI at 6 months

	Beta	SE	95 % CI	p
intercept	63.52	19.49	4.95 - 67.61	0.024
gender (female)	12.72	3.71	5.29 - 20.14	0.001
perceived health	4.54	2.13	0.27 - 8.81	0.038
2-min walk (m)	0.20	0.05	0.10 - 0.31	<0.001
emotional well-being	0.22	0.12	^−^0.02 - 0.45	0.066
very low income	−15.7	6.99	^−^29.69 - ^−^1.72	0.028

Adj R^2^ = 0.47

**Table 4:** Multivariate model of RNLI at 9 months

	Beta	SE	95 % CI	p
intercept	45.70	19.68	^−^4.27 – 51.51	0.095
gender (female)	9.41	3.27	2.86 – 15.96	0.006
perceived health	3.68	2.50	^−^1.34 – 8.70	0.147
2-min walk (m)	0.22	0.05	0.118 – 0.31	<0.001
emotional well-being	0.36	0.12	0.127 – 0.59	0.003
very low income	−10.30	5.96	^−^22.25 – 1.66	0.090

Adj R^2^ = 0.58

**Table 5:** Multivariate model of RNLI at 12 months

	Beta	SE	95 % CI	p
intercept	42.52	17.82	8.95 – 69.41	0.012
gender (female)	8.92	3.34	2.23 – 15.60	0.010
perceived health	0.56	1.97	^−^3.38 – 4.50	0.778
2-min walk (m)	0.18	0.05	0.08 – 0.27	0.001
emotional well-being	0.41	0.12	0.17 – 0.64	0.001
very low income	−14.45	6.86	^−^28.19 – ^−^0.71	0.040

Adj R^2^ = 0.52

**Table 6:** Multivariate model of RNLI at 18 months

	Beta	SE	95 % CI	p
intercept	40.81	19.39	^−^15.98 – 46.19	0.333
gender (female)	9.53	3.60	2.28 – 16.78	0.011
perceived health	4.28	2.17	^−^0.08 – 8.65	0.054
2-min walk (m)	0.12	0.04	0.03 – 0.21	0.008
emotional well-being	0.43	0.11	0.21 – 0.66	<0.001
very low income	^−^1.80	6.86	^−^14.76 – 11.16	0.781

Adj R^2^ = 0.57

**Table 7:** Multivariate model of RNLI at 24 months

	Beta	SE	95 % CI	p
intercept	36.83	21.99	^−^10.10 – 63.44	0.150
gender (female)	8.93	4.20	0.42 – 17.45	0.040
perceived health	1.69	2.85	^−^4.09 – 7.48	0.556
2-min walk (m)	0.10	0.06	^−^0.10 – 0.22	0.073
emotional well-being	0.47	0.14	0.19 – 0.74	0.002
very low income	^−^3.88	8.07	^−^20.26 – 12.50	0.634

Adj R^2^ = 0.50

Table 8 shows the results from the GEE model. The effect of very low-income neighbourhood is both substantively strong and statistically significant – those in very low-income neighbourhoods had average RNLI scores approximately 13 points lower (p = .003). The effect of time was not significant, but all of the other predictors were. Women had higher RNLI scores, B = 8.785, SE = 2.573, p = .001. Better perceived health was associated with higher RNLI scores, B = 2.184, SE = .936, p = .020. Higher values on the two-minute walk variable were also associated with higher RNLI scores, B = .120, SE = .028, p < .001. Finally, improved emotional well-being was associated with higher RNLI scores, B = .370, SE = .071, p < .001.

**Table 8:** GEE Model of RNLI

Parameter	B	Std. Error	95 % Wald Confidence Interval		Hypothesis Test		
			Lower	Upper	Wald Chi-Square	df	Sig.
(Intercept)	33.63	5.78	30.06	63.42	30.17	1	< .001
Gender: Female	8.79**	2.57	3.74	13.83	11.66	1	.001
Perceived Health	2.18*	0.94	−4.02	-.35	5.45	1	.020
Two Minute Walk	0.12**	0.03	.07	.17	18.72	1	< .001
Emotional Well-Being	0.37***	0.07	.23	.51	26.99	1	< .001
Very Low Income	−12.98**	4.38	−21.57	−4.39	8.76	1	.003
Time	.78	0.50	-.21	1.76	2.39	1	.123

Note. Exchangeable covariance structure. ***p < .001. **p < .01. *p < .05

As Table 2 showed that RNLI improved with time for those from higher income neighbourhoods but not for those from very low income neighbourhoods, this suggests that there may be an income by time interaction. This possibility was tested in a separate post hoc GEE, but the interaction term was not significant (p = .405).

## Discussion

There is evidence that very low neighbourhood income is associated with greater risk of stroke, greater risk of more severe stroke, and lower levels of care [17–19]. This study demonstrates that individuals within very low income neighbourhoods are also more likely to have difficulty participating in personally valued activities, even when the level of impairment and other factors related to participation are taken into account. This is consistent with predictions based on Bernard’s conceptualization of the relationship between health inequities and neighbourhood income [15]. In addition, our results suggest that neighbourhood income may be an important contextual feature to consider when conceptualizing the relationship between impairment and participation. Our results may also suggest issues related to personal low income, since people living in very low income neighbourhoods have a higher probability of personal low income. Limited income could directly restrict individuals from activities that involve costs (such as shopping, dining, club membership) while contributing to problems accessing free activities (such as affording transportation). From a psychological perspective, higher income may provide a needed buffer against the stresses created by the illness; without such a buffer, latent psychological problems, such as depression, may emerge, creating further barriers to engagement in valued activity [35].

Among the important limitations of this study are a relatively small sample size and a small number of participants from very low income neighbourhoods. This may have led to unstable estimates. As well, our inclusion criteria that barred those with more severe language and cognitive impairment from participating in the study. This later issue likely created a bias towards the inclusion of relatively high functioning stroke survivors. Whether neighbourhood income has a similar impact on the participation of those with higher levels of disability, particularly severe cognitive and language problems, is not known. In addition, the recruitment rate for the study was relatively low at 34.9 % [27]. While this is not uncommon for this type of study [6], it does introduce a potential bias if eligible lower or higher income neighbourhood patients who declined participation had different profiles of participation than the lower or higher income patients who were recruited. Finally, we did not inquire about participation in personally valued activities prior to the stroke. We do not know how much of the difference in participation between the two groups predated the stroke.

These results can be considered only preliminary due to the small number of participants from very low income neighbourhoods. Further research, with a larger sample is warranted. However, the results are quite striking, and indicate that from a clinical perspective, neighbourhood income should be considered when designing interventions to promote participation post stroke.

## Conclusion

Low neighbourhood income has a well-documented impact on stroke incidence, severity, mortality and care. Our findings provide preliminary indication that stroke survivors from very low income neighbourhood are also likely to experience greater difficulty with participation during the first two years post stroke.

## Abbreviations

Cdn: Canadian
FIM™: Functional impairment measure
RNLI: Reintegration to normal living index
